# Evaluation of the efficacy of the SARS-CoV-2 vaccine additional and booster doses in immunocompromised patients with multiple sclerosis: the COVACiMS study

**DOI:** 10.1007/s00415-025-12991-8

**Published:** 2025-03-25

**Authors:** Filipa Ladeira, Claudia Nobrega, João Cerqueira

**Affiliations:** 1 Multiple Sclerosis Center of Integrated Responsibility, Hospital de Santo António Dos Capuchos, Unidade Local de Saúde São José, Lisbon, Portugal; 2 Centro Clínico Académico de Lisboa, Lisbon, Portugal; 3 ICVS/3B’s- PT Government Associate Laboratory, Life and Health Sciences Research Institute (ICVS), Braga, Portugal; 4 Neurology Department, Hospital de Braga and 2CA – Clinic Academic Centre Braga, School of Medicine, University of Minho, Braga, Portugal

**Keywords:** Multiple sclerosis, COVID-19, Vaccination, Humoral immune response; Cellular immune response

## Abstract

Studies evaluating COVID-19 primary vaccination with two vaccines reported a blunt response in Multiple Sclerosis (MS) patients under anti-CD20 and sphingosine-1-phosphate (S1P) modulators. An extended primary vaccination (EPV) was recommended in immunosuppressed MS patients. Data on the effectiveness of the EPV and subsequent booster dose are limited. A prospective cohort study (n = 270) was conducted to evaluate the humoral and cellular immunogenicity of the EPV scheme in immunocompromised MS patients (i.e., treated with anti-CD20, S1P modulators, natalizumab, teriflunomide, or dimethyl fumarate) vs. regular primary vaccination in non-treated patients - primary course (PC) cohort. The effect of a subsequent booster dose was also assessed - first booster (FB) cohort . The seroconversion rates were 55% and 56% in anti-CD20 and 75% and 67% in S1P modulators group in PC and FB cohort, respectively, and 100% in the remaining groups. A positive SARS-CoV-2 Spike T-spot was observed in 22% of patients under S1P modulators in PC cohort and 67% in FB cohort; the remaining groups had 75% or more. Similar rates of breakthrough infection were observed in both groups vs. controls. Compared to non-treated MS patients, immunosuppressed patients under anti-CD20 and S1P modulators drugs receiving EPV scheme or booster dose still present lower protection rates to SARS-CoV-2.

**Supplementary Information:**

The online version contains supplementary material available at 10.1007/s00415-025-12991-8.

## Introduction

A Severe Acute Respiratory Syndrome (SARS) associated with SARS-CoV-2 emerged in December 2019, causing over 7 million deaths worldwide [1]. By the end of 2020, vaccines were developed at an unprecedented speed [2]. Immunogenicity studies on a two-dose COVID-19 vaccine regimen showed lower antibody/neutralization titers in moderate-to-severely immunocompromised individuals compared to non-immunocompromised individuals across various diseases. [3]Additionally, data suggested a higher risk of breakthrough infection in immunocompromised patients following a two-dose vaccination [4]. In multiple sclerosis (MS) patients on immunosuppressive disease-modifying therapies (DMTs), similar observations of reduced immunogenicity [5–9] and increased breakthrough infection risk were reported [10, 11], particularly with anti-CD20 and sphingosine-1-phosphate (S1P) receptor modulators. Factors like shorter time since the last infusion and lower B-cell repopulation were linked to reduced humoral response in anti-CD20 patients, while lymphopenia was associated with lower vaccine immunogenicity in S1P modulator patients. [5, 8, 9, 12, 13] In response, additional doses to the primary vaccination course were recommended by immunization guidelines in August–October 2021, as part as the primary vaccination scheme - extended primary vaccination (EPV). [14–16] MS patients on DMTs, except for interferons or glatiramer acetate, were eligible for EPV according to the Portuguese guidelines [17]. Due to waning immune responses observed in healthy individuals, [18, 19] a booster dose was recommended 3–6 months after the primary vaccination or SARS-CoV-2 infection. Initially, only immunocompromised individuals received boosters in Portugal (by September 2021), but this was extended to all adults 2 months later. [20] The effectiveness of EPV and booster doses in immunosuppressed MS patients on DMTs remains uncertain. This issue is critical as immunocompromised individuals are more likely to experience severe COVID-19 and prolonged SARS-CoV-2 infection, facilitating viral evolution. [21–27]

This study aimed to assess the immunogenicity of the EPV (primary vaccination with an additional dose) in MS patients under DMT, excluding interferons or glatiramer acetate, compared to the regular primary vaccination (RPV; primary vaccination without an additional dose) in untreated patients (not eligible for EPV).

With the progression of the SARS-CoV-2 pandemic and vaccination schemes in Portugal (Supplementary Table 1), the study's objectives expanded to include evaluating the immunogenicity of a single COVID-19 vaccine booster doses in subjects under various DMT and reporting breakthrough COVID-19 rates under different vaccination protocols.

## Methods

### Study design

**Fig. 1:**
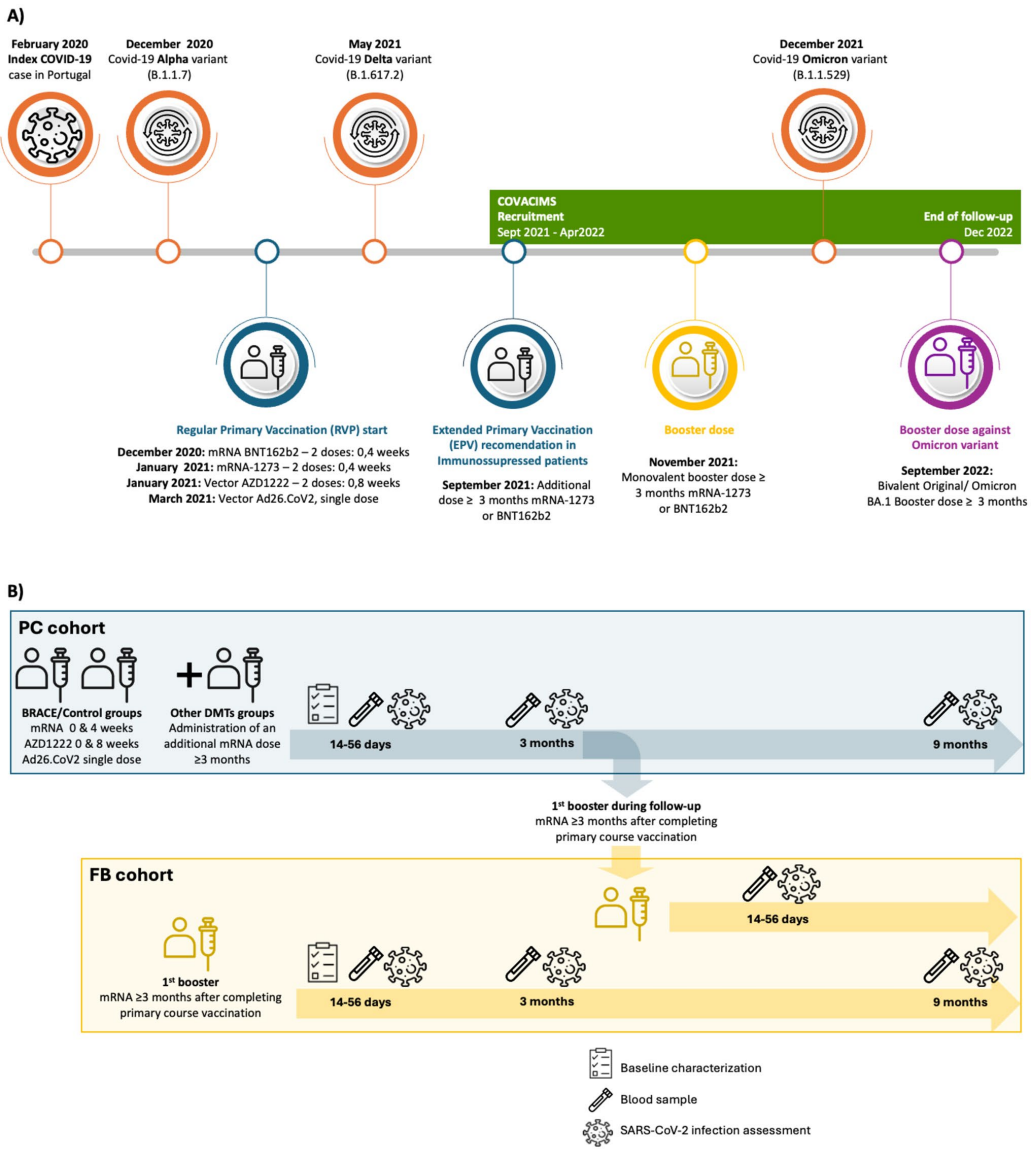
COVACIMS setting and procedures. **A** Evolution of COVID-19 pandemic and vaccination recommendations in Portugal between 2020 and 2022 vs. timing of COVACiMS study. **B** COVACiMS study design. The study is composed of two cohorts: the primary vaccination course (PC) cohort, and the first booster dose (FB) cohort. Some patients from the PC cohort received a first booster dose during follow-up and were requested to provide an additional blood assessment. These patients were latter included in the FB cohort allowing paired analysis of the data. Demographic and clinical data were collected at baseline (i.e., at study entry), and blood collected at the defined time points

A multicenter prospective cohort study on MS patients receiving the COVID-19 vaccine was conducted from September 15, 2021, to April 21, 2022, across eight Portuguese hospitals, with follow-up ending in December 2022 and data collection completed by February 2023 (Fig. 1A). According to local guidelines at the time [17], the control group (non-treated MS) and the BRACE (Betaferon®, Rebif®, Avonex®, Copaxone®, Extavia®) treated group received either two mRNA vaccines, two AZD1222 doses, or one Ad26.CoV2 viral vector vaccine (RPV) for a complete primary course. For other DMT-treated groups, the primary course included two mRNA vaccines, two AZD1222 vaccines, or one Ad26.CoV2 viral vector vaccine, followed by an additional mRNA dose (EPV). Any doses after this primary course were considered boosters (Supplementary Table 1). Only original monovalent COVID-19 vaccines were analyzed; patients receiving bivalent formulations (Original and Omicron BA.4/BA.5) during follow-up were censored at inoculation time. To assess the effectiveness of the primary vaccination course and the first booster dose, patients were enrolled 14–56 days post-primary vaccination - primary course (PC) cohort - or first booster dose - first booster (FB) cohort - and underwent blood tests to evaluate specific B- and T-cell responses to the SARS-CoV-2 spike protein. PC patients receiving a booster during the study provided an additional blood sample 14–56 days post-booster and joined the FB cohort; failure to do so resulted in being lost to follow-up (Fig. 1B). Breakthrough COVID-19 infection was monitored at 3 and 9 months (± 1 week) post-primary vaccination (PC cohort) or post-booster (FB cohort).

### Ethics

This study adhered to the ICH Harmonized Tripartite Guidelines for Good Clinical Practice and the Declaration of Helsinki's ethical principles. The protocol received approval from Ethics Committees and Administration Boards of all centers. Written informed consent was obtained prior to any study procedures.

### Participants

Eligible subjects had completed a primary vaccination scheme or a first booster dose within the previous 14–56 days, were over 18 years old, and provided written informed consent. They had clinically isolated syndrome or MS diagnosed per the McDonald Criteria 2017, and a complete vaccination scheme by the time of enrollment (June 2021 to March 2022, with the last vaccine dose by March 01, 2022). Exclusion criteria included treatment for neoplastic conditions or other immunosuppressant/immunomodulator treatments at any SARS-CoV-2 vaccine dose date.

### Study procedures

Baseline data, including demographic, MS clinical data, treatment, COVID-19 infection, and vaccination details, were collected from patient records through a structured questionnaire (Supplementary data). Blood assessments at enrollment involved immunophenotyping (CD20; CD8 and CD4 T-cell counts) and evaluating humoral (spike and nucleocapsid-specific IgG) and cellular responses (spike and nucleocapsid-reactive T cells). For those receiving a first booster, the same blood analysis was repeated 14–56 days post-booster. During follow-up visits, data on current DMT, COVID-19 occurrences, and PCR confirmations since the last visit were collected via a structured questionnaire and confirmed through electronic health records. Blood assessments were also conducted to detect undiagnosed SARS-CoV-2 infections by evaluating SARS-CoV-2 nucleocapsid-specific humoral and cellular responses. Vaccination regimens were classified as heterologous (mRNA and viral vector vaccine combination) or homologous (single vaccine type, either mRNA or viral vector). Participants were categorized into treatment groups based on their treatment at the first vaccine dose: control (no DMT); BRACE natalizumab; first-line immunomodulators other than BRACE (FLIM), including teriflunomide or dimethyl fumarate; anti-CD20 agents (rituximab, ocrelizumab, ofatumumab); S1P modulators (fingolimod, siponimod); and IRT (cladribine, alemtuzumab). The group categorization was primarily based on the vaccination scheme at the time of recruitment, namely the number of vaccines included in the primary vaccination (1–2 vaccine doses in non-treated and BRACE-treated patients -RPV vs 2–3 vaccine doses in patients under the remaining DMT- EPV). The groups were divided further by the presumed DMT impact on vaccine immunogenicity, in accordance with the previous reports [28].

### Laboratory procedures

Details on laboratory procedures, sample processing, isolation of peripheral blood mononuclear cells, T- and B-cell counts, ELISPOT assay, and SARS-CoV-2 serology are provided in the Supplementary material.

### Outcomes

We evaluated B- and T-cell responses to the SARS-CoV-2 spike protein following a complete primary vaccination course (PC) or the first booster dose (FB) by DMT. Outcomes included seroconversion rates (based on SARS-CoV-2 spike-specific IgG cut-off values; supplementary Table 2), SARS-CoV-2 spike-specific IgG titers, reactivity rates of the SARS-CoV-2 Spike T-spot assay, and spike-reactive T-cell counts. For breakthrough COVID-19 outcomes, we tracked infections occurring 14 days post-vaccination, confirmed via self-report, electronic health records, and/or laboratory results detecting humoral and cellular responses specific to the SARS-CoV-2 nucleocapsid protein.

### Statistical analysis

Categorical variables were described by absolute and relative frequencies and 95% confidence intervals and compared using McNemar’s χ2 test. Continuous variables were described by mean and SD or median and IQR and compared using Student’s t test or Wilcoxon’s tests. Comparative analyses of primary vaccination and first booster dose immunogenicity between treatment and control groups were performed. Unadjusted and adjusted analyses using regression models were attempted but showed poor fits (results not shown). The association between CD20 + , CD3 + , CD3 + CD4 + , and CD3 + CD8 + counts and primary vaccination immunogenicity was tested for patients on anti-CD20 agents and S1P modulators. Paired comparisons in patients on these treatments were conducted before and after their first booster to assess improvements in immunogenicity outcomes. Breakthrough and non-breakthrough infection rates were calculated for each treatment group in the PC and FB cohorts, adjusted for follow-up time, taking the date of the last vaccination dose as a reference. Kaplan–Meier estimators were used to calculate and compare the risk of breakthrough infection across study groups. All tests were two-sided with a 5% significance level. Normality was tested using Shapiro–Wilk test. Analyses were performed using R v4.4.1.

## Results

### Study population

The analyses included 270 patients: 211 in the primary cohort (PC) and 146 in the first booster (FB) cohort; 87 patients were included in both cohorts due to vaccination status changes during the study. Table 1 details patients’ demographic and clinical characteristics at study entry by cohort. All S1P modulator patients were treated with fingolimod; of the 31 anti-CD20 patients in the PC group, 3 were on rituximab and the rest on ocrelizumab; all anti-CD20 patients in the FB cohort were treated with ocrelizumab. Most primary course vaccinations were completed with homologous mRNA vaccines (Table 1). All additional and booster doses were mRNA vaccines, administered on average 5 months (153.4 ± 47 days) after the last administration or 6 months (199 ± 44 days) after the primary vaccination course.

**Table 1:** Demographic and clinical characteristics at baseline (i.e., at study entry)

	PC cohort (*n* = 211)	FB cohort (*n* = 146)
Female sex, *n* (%)	139 (67.8)	90 (61.6)
Age (years), Mean ± SD	43.2 ± 11.1	45.54 ± 10.5
MS type, *n* (%)		
Clinically isolated syndrome	2 (0.9)	2 (1.4)
Primary progressive MS	15 (7.1)	17 (11.6)
Relapsing–remitting MS	178 (84.4)	117 (80.1)
Secondary progressive MS	16 (7.6)	10 (6.9)
Disease duration (years), median (IQR)	7 (11)	6 (12)
EDSS, median (IQR)	2 (3.0)^a^	2 (2.5)^b^
Treatment groups, *n* (%)		
Control	6 (2.8)	38 (26.0)
BRACE	11 (5.2)	32 (21.9)
Natalizumab	66 (31.3)	22 (15.1)
FLIM	58 (27.4)	23 (15.8)
Anti-CD20	29 (13.7)	18 (12.3)
S1P	20 (9.5)	7 (4.8)
IRT	21 (10.0)	6 (4.1)
Vaccination regimen, *n* (%)		
Homologous	190 (90.1)	121 (82.9)
^c^Heterologous	21 (9.9)	25 (17.1)

^a^Missing: *n* = 4 (1.9%); ^b^Missing: *n* = 5 (3.4%); ^c^all mRNA vaccines

*BRACE* Betaferon®, Rebif®, Avonex®, Copaxone®, Extavia®, *EDSS* Expanded Disability Status Scale, *FLIM* first-line immunomodulators other than BRACE, *IQR* interquartile range, *IRT* immune reconstitution treatments, *MS* multiple sclerosis, *S1P* sphingosine-1-phosphate, *SD* standard deviation

**Fig. 2:**
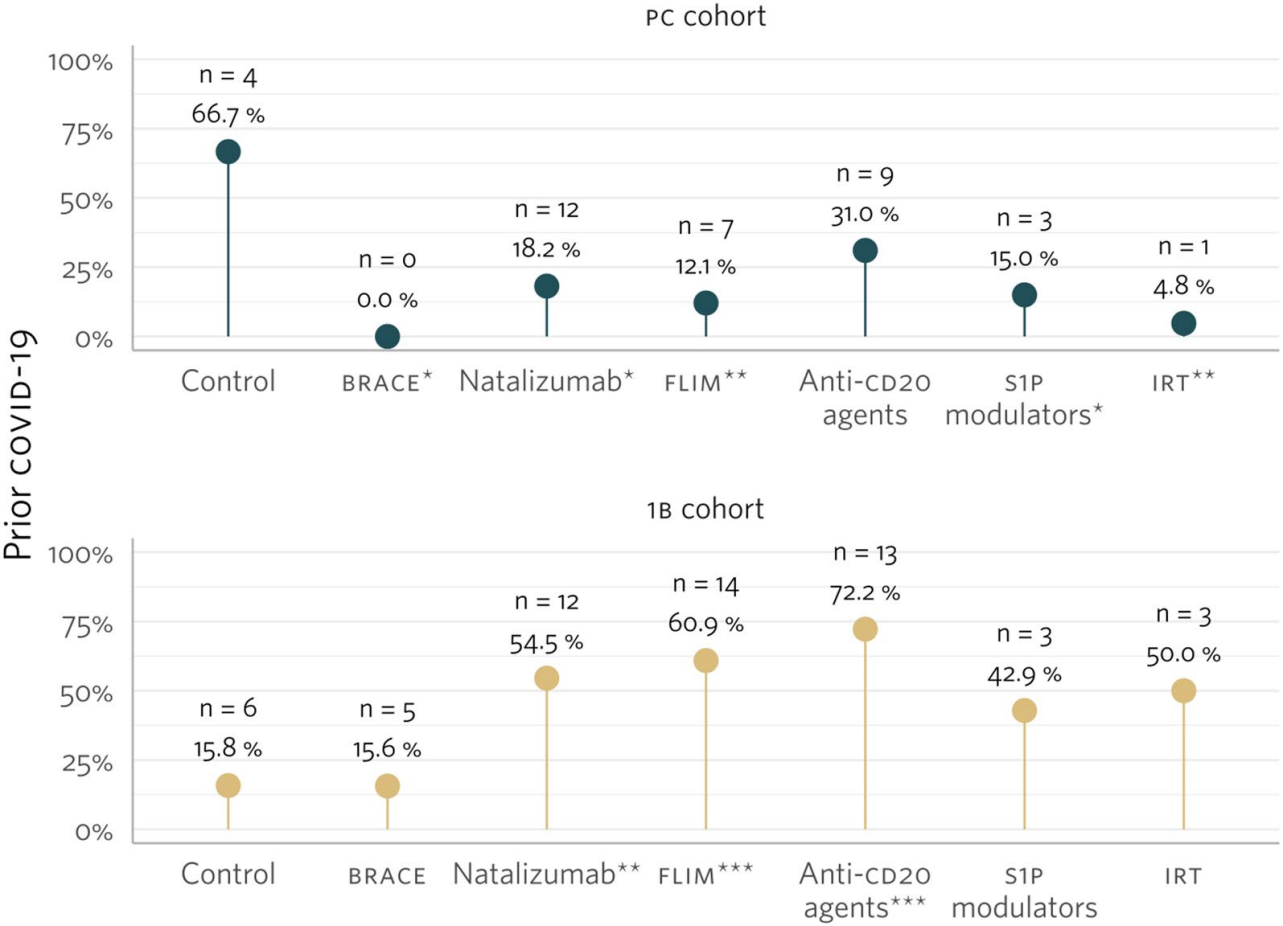
Percentage of prior SARS-CoV-2 infection, per study cohort, and treatment group: **A** primary course (PC) cohort; **B** first boost (FB) cohort. The diagnosis of SARS-CoV-2 infection was either reported by the patient or ascertained from the electronic health records. The assessment of undiagnosed infection was conducted by evaluating SARS-CoV-2 nucleocapsid-specific humoral and cellular responses. The percentage of prior SARS-CoV-2 infection in the control group was compared to the DMT groups using McNemar’s χ2 test; statistically significant differences (*p* value < 0.050) where represented by * for *p* value =] 0.050; 0.01]; ** for *p* value =]0.01; 0.001]; and *** for p value < 0.001

SARS-CoV-2 infection before completing the primary vaccination was documented in 36 patients (17.1%), and in 56 patients (38.4%) before the first booster. Infection rates varied by treatment group: in the PC cohort, prior COVID-19 was more common in the control group compared to all treatment groups except anti-CD20. In the FB cohort, prior COVID-19 was less common in the control group compared to all treatment groups except BRACE, S1P modulators, and IRT groups (Fig. 2).

### Impact of primary course vaccination on humoral and cellular immune response by DMT

**Fig. 3:**
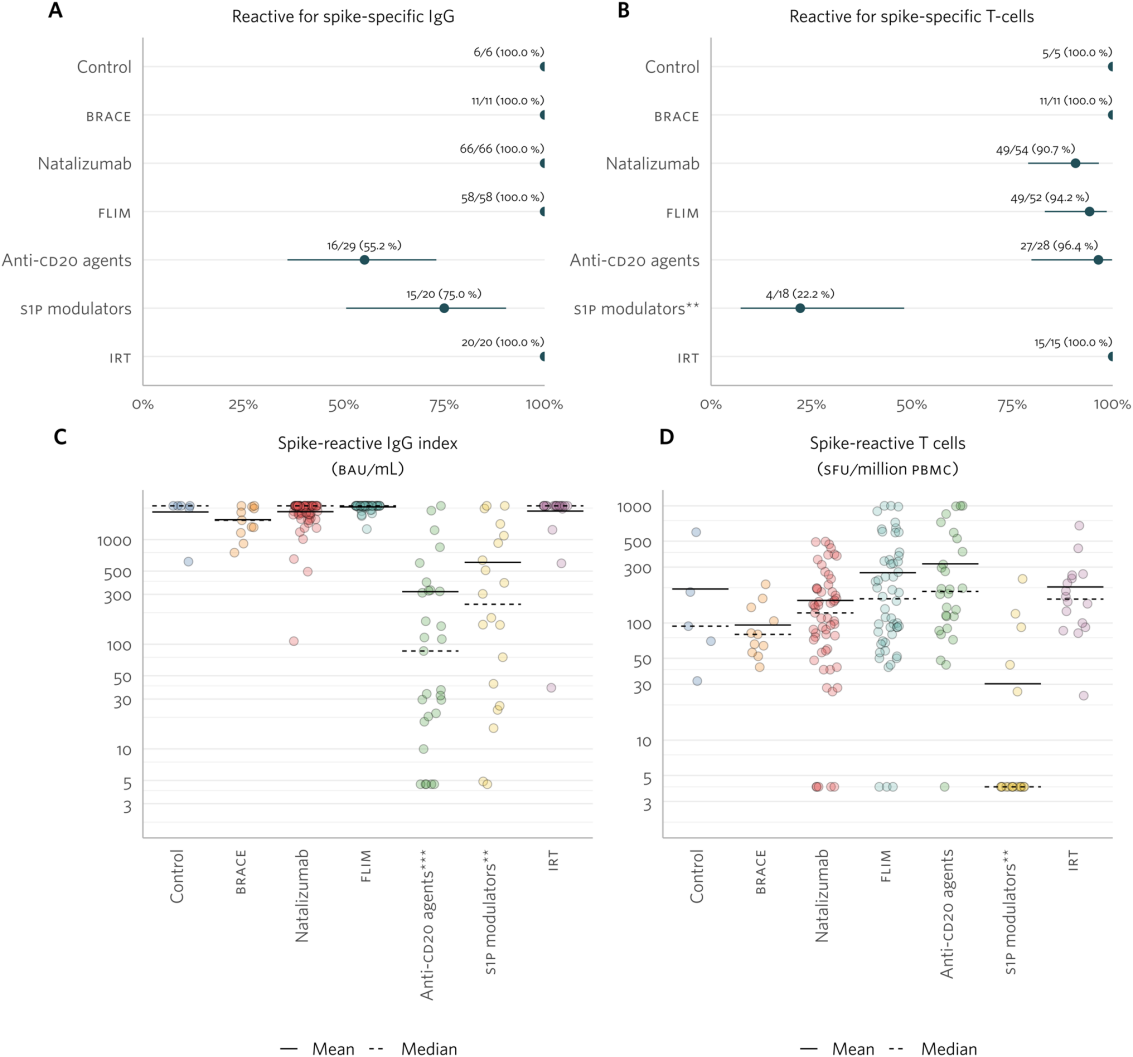
SARS-CoV-2-specific humoral and cellular response in the primary course (PC) cohort, per treatment group. Percentage of patients with spike-specific immunoglobulin G (IgG; **A**) and with spike-specific T cells (**B**). Absolute quantification of spike-specific IgG in the blood (indexes; **C**), and number of spike-specific T cells per million of peripheral blood mononuclear cells (PBMCs, **D**). Statistically significant differences (*p* value < 0.050) were represented by * for p value =]0.050; 0.01]; ** for *p* value =]0.01; 0.001]; and *** for *p* value < 0.001

In the PC cohort, all control, BRACE, natalizumab, FLIM, and IRT group patients seroconverted post-primary vaccination. IgGs reactive to the SARS-CoV-2 spike protein were observed in 55% of the anti-CD20 group and 75% of the S1P modulators group, though differences were not significant compared to the control group (Fig. 3A). Over 90% of patients had a positive SARS-CoV-2 Spike T-spot assay in all groups except the S1P modulators, where 22% had a positive result (Fig. 3B). The mean spike-specific IgG index was over three times higher in the control group vs the anti-CD20 or S1P modulators groups (Fig. 3C). Mean spike-reactive T-cell counts were over three times lower in the S1P modulators group (Fig. 3D). Due to low variability and potential bias from upper and lower artificial bounds, no regression analysis adjusted for prior COVID-19 was conducted.

For anti-CD20 and S1P modulators groups in the PC cohort, we examined associations between SARS-CoV-2 spike protein reactiveness (i.e., individuals with spike-specific IgG or spike-reactive T cells) and patients’ lymphocyte counts (i.e., B cells—CD20 + ; CD4 and CD8 + T cells–CD3 + CD8- and CD3 + CD8 +). Patients on S1P modulators with a reactive spike-specific IgG response had significantly higher CD8 + T-cell counts than non-reactive patients (Table 2). No other lymphocyte counts’ associations with SARS-CoV-2 spike protein reactiveness were found.

**Table 2:** Lymphocyte counts vs reactiveness to SARS-CoV-2 spike protein in PC cohort, in anti-CD20 and S1P modulators groups

	Spike-specific IgG index					Spike-reactive T-cell counts				
	Reactive		Non-reactive		*p*	Reactive		Non-reactive		*p*
	*n*	Median ± IQR	n	Median ± IQR		n	Median ± IQR	n	Median ± IQR	
Anti-CD20 agents										
B cells	9	59.0 ± 38.0	6	80.0 ± 85.8	0.456	14	59.5 ± 56.0	1	171.0	0.267
CD4 + T cells	16	703.5 ± 285.5	12	675.0 ± 315.0	0.347	27	700.0 ± 238.0	1	566.0	0.643
CD8 + T cells	16	378.5 ± 309.0	12	217.0 ± 216.0	0.082	27	339.0 ± 315.0	1	208.0	0.643
S1P modulators										
B cells	13	55.0 ± 199.0	5	37.0 ± 13.0	0.117	2	46.0 ± 16.0	14	49.3 ± 20.2	0.933
CD4 + T cells	15	177.0 ± 168.0	5	69.0 ± 43.0	0.060	4	186.0 ± 155.0	14	128.0 ± 134.0	0.710
CD8 + T cells	15	261.0 ± 305.0	5	99.0 ± 27.0	**0.021**	4	245.5 ± 300.0	14	202.0 ± 148.0	0.873

*IQR* interquartile range, *S1P* sphingosine-1-phosphate

### Impact of disease-modifying therapies on the efficacy of a first booster dose

**Fig. 4:**
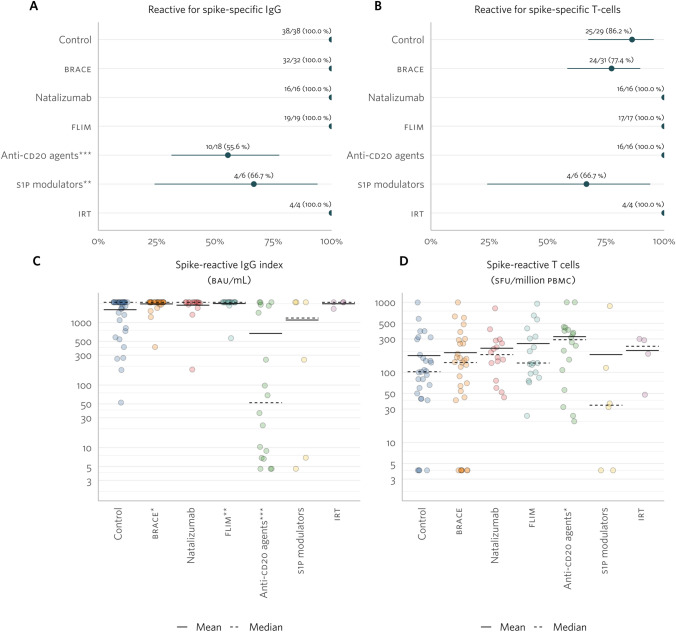
SARS-CoV-2-specific humoral and cellular response in the first booster (FB) cohort, per treatment group. Percentage of patients with prior SARS-Cov-2 infection (**A**), with spike-specific immunoglobulin G (IgG; **B**) and with spike-specific T cells (**C**). Absolute quantification of spike-specific IgG in the blood (indexes; **D**), and number of spike-specific T cells per million of peripheral blood mononuclear cells (PBMCs, **E**). Statistically significant differences (*p* value < 0.050) were represented by * for *p* value =]0.050; 0.01]; ** for *p* value =]0.01; 0.001]; and *** for *p* value < 0.001

Seroconversion rates in the FB cohort mirrored those in the PC cohort, with all patients in the control, BRACE, natalizumab, FLIM, and IRT groups seroconverting. The seroconversion rates in the SP1 modulators and anti-CD20 groups were lower than in the control group (67% and 56%, respectively; Fig. 4A). However, the proportion of patients with a positive spike-reactive T-spot assay was consistent across control and treatment groups, including S1P (Fig. 4B).

The mean spike-specific IgG index and spike-reactive T-cell counts in the FB cohort by treatment group are shown in Fig. 4. Patients on anti-CD20 and S1P modulators had a mean spike-specific IgG index approximately twofold lower than the control group (Fig. 4C). The mean spike-reactive T-cell counts were twice as high in the anti-CD20 group (Fig. 4D). No model adjusted for prior COVID-19 was attempted due to data artificial bounds.

### Paired comparisons before and after booster dose in anti-CD20 and S1P modulator groups

After the first booster dose, some patients acquired a spike-specific immune response absent after primary vaccination. However, due to the small sample size, no statistical differences were noted in seroconversion rates or spike-reactive T-cell response post-booster. The magnitude of response, indicated by the spike-specific IgG index and spike-reactive T-cell counts, did not significantly change with the booster dose (Table 3). Notably, some patients who lacked a detectable T-cell response after primary vaccination showed a de novo response post-booster.

**Table 3:** Immunogenicity following primary vaccination vs first booster paired comparison, in patients under anti-CD20 and S1P modulators

	Percentage positive				Absolute values			
	n	PC cohort	FB cohort	*p*	n	PC cohort	FB cohort	*p*
		%	%			Median ± IQR	Median ± IQR	
Spike-specific IgG								
Anti-CD20	18	61.1	55.6	1.000^c^	18	139 ± 522.0	52.2 ± 1740.0	0.979^a^
S1P modulators	5	60.0	60.0	–	5	286.5 ± 456.0	893.1 ± 1110.0	0.191^b^
Spike-reactive T cells								
Anti-CD20	15	93.3	100.0	1.000^c^	17	356.5 ± 347.0	330.1 ± 294.0	0.661^b^
S1P modulators	4	0.0	50.0	0.480^c^	5	4.0 ± 0.0	32.0 ± 112.0	0.181^a^

^†^Mean ± Standard deviation; aWilcoxon rank sum test with continuity correction; b: Student’s *t* test for paired samples; c: Mc Nemar’s Chi-squared test; *IQR* interquartile range, *S1P* sphingosine-1-phosphate

### Assessment of breakthrough SARS-CoV-2 infection

Breakthrough and non-breakthrough infection rates were calculated for each treatment group in the PC and FB cohorts, adjusted for follow-up time (Table 4). There were 26 breakthrough infections in the PC cohort and 21 in the FB cohort, with an average of 38 days between the last vaccination dose and a breakthrough infection (range: 15–125 days). The cumulative incidence of breakthrough COVID-19 was 0.1691 infections per patient-year in the PC cohort and 0.3496 in the FB cohort (Table 4).

**Table 4:** Rates of breakthrough infections in the PC and FB cohorts, globally and per disease-modifying therapy

	Total follow-up (patient-year)		Breakthrough COVID-19 (infections per patient-year)			
	PC cohort	FB cohort	PC cohort		FB cohort	
			*n*	rate	*n*	rate
**Global**	153.77	60.07	26	0.17	21	0.35
Control	3.24	24.53	1	0.31	4	0.16
BRACE	5.94	25.56	0	0.00	4	0.16
Natalizumab	47.37	3.26	11	0.23	3	0.92
FLIM	43.98	2.85	4	0.09	5	1.75
anti-CD20	21.06	2.12	7	0.33	3	1.42
S1P modulators	15.29	0.97	3	0.20	1	1.03
IRT	16.89	0.78	0	0.00	1	1.28

*BRACE* Betaferon®, Rebif®, Avonex®, Copaxone®, Extavia®, *FLIM* first-line immunomodulators other than BRACE, *IRT* immune reconstitution treatments, *S1P* sphingosine-1-phosphate

Although immunogenicity following vaccination in the anti-CD20 agents and S1P modulators groups was compromised to some extent vs. the control group, the breakthrough COVID-19 rate was similar (Table 4).

## Discussion

To our knowledge, this is the first study evaluating the original monovalent COVID-19 vaccination course including an additional and a booster dose in MS immunosuppressed patients, as recommended by the WHO in October 2021 [16]. Also, this is the first study evaluating the immunogenicity of COVID-19 vaccines in MS patients in Portugal.

We evaluated the qualitative and quantitative B- and T-cell response to the COVID-19 EPV series with an additional vaccine dose in immunosuppressed patients (i.e., all DMT-treated groups, excluding BRACE) vs. the RPV scheme without an additional dose in non-treated MS patients (control group). In accordance with previous reports, the group categorization was based first on the number of vaccine doses comprised in the primary vaccination scheme, and second on the presumed impact of DMT on overall vaccine immunogenicity. [28]Although in the literature, the BRACE group showed similar vaccination immunogenicity to untreated MS patients, we analyzed these groups separately. This decision was made mostly based on the fact that the PC cohort had a significantly different incidence of prior COVID-19, and combining the BRACE group with treatment-naïve individuals would have increased group’s heterogeneity. Also, we analyzed the response of the first booster dose—2nd/3rd dose in control group vs. 3rd/4th dose in patients under DMTs other than BRACE. Overall, vaccine immunogenicity was mostly affected in the S1P modulators and anti-CD20 groups, compared to control group. Paired comparisons before and after the booster dose in S1P modulators and anti-CD20 treated patients, despite not revealing a significant improvement in any immunogenicity parameter studied, demonstrated that some patients without previous SARS-CoV-2 spike-specific T-cell mounted a de novo response.

Our findings align with the previous studies on the blunted response to the COVID-19 vaccine in patients under anti-CD20 and S1P modulators [29–31]

However, previous meta-analysis identified a seroconversion rate following RPV of 36% in patients under anti-CD20 drugs and 60% in patients under S1P modulators, while on our work, the seroconversion rate was about 50% and 75%, respectively, suggesting a benefit of EPV in these patients. As previously reported, the effect of booster doses in patients under anti-CD20 and S1P modulators was modest and the immune response to booster doses was inferior to the observed in non-treated patients, even though the latter received an inferior number of vaccine doses [32–34].

We assessed the relation between subset lymphocyte count and immunogenicity of primary vaccination in patients under anti-CD20 and S1P modulators groups and found that CD8 + T-cell counts were higher in patients that seroconverted following primary vaccination. CD8 + T-cell counts have been associated with plasmoblasts’ expansion and anti-SARS-CoV-2 antibody secretion [35], and similar findings in MS patients were previously reported. [36]

Our results suggest that the breakthrough COVID-19 rate was not higher in patients under anti-CD20 and S1P modulators compared to the remaining. This might imply that the vaccination scheme recommended by the WHO in October 2021[16] -EPV followed by booster in susceptible individuals, resulted in a better protection of vulnerable groups and in balanced rates of breakthrough COVID-19 infection compared to non-treated patients. Nevertheless, several factors that may contribute to the infection rate were not evaluated (age, specific viral variants) which limits data interpretation.

Our findings support the recommendations on the use of an additional vaccine dose as part of primary vaccination scheme—EPV, followed by booster doses. Even though the use of the EPV course was not sufficient to produce an immune response similar to the RPV in non-treated patients, we observed higher seroconversion rates in patients under anti-CD20 and S1P modulators compared with the previous reports. Additionally, booster doses generated a de novo T-cell response in some patients that were previously unresponsive. The observation of a similar rate of breakthrough COVID-19 in treated and untreated patients is an additional supportive factor, but the small sample size might limit the robustness of this result.

Our study has some limitations, related to the fact that this is a real-world study, performed in a time-period with high SARS-CoV-2 infection rates. This setting created a scenario of dynamic conditions in COVID-19 vaccination schemes leading to some groups having small sample sizes that, together with artificial boundaries imposed in some of the assays (e.g., spike-specific IgG and spike-reactive T-cell quantification), hampered multivariate analysis. Prior SARS-Cov-2 infection might have contributed to the observed immunogenicity and our analysis was not adjusted to this cofactor. However, because, in the PC cohort, previous COVID-19 was more frequent in the control group, one would expect to amplify differences in immune response vs. the anti-CD20 and S1P modulators groups, and no statistical difference in the qualitative humoral and cellular response was observed. On the contrary, in the FB cohort, the qualitative response was significantly impaired in the anti-CD20 and S1P modulators groups despite the higher frequency of prior COVID-19 in these groups.

The main strength of the study is the characterization of both B- and T-cell response following primary vaccination and booster doses, and the subsequent longitudinal follow-up and registration of breakthrough COVID-19, both symptomatic and laboratory-confirmed.

In conclusion, this study suggests that there is a benefit in using EPV including an additional dose in patients under anti-CD20 and S1P modulators, since the seroconversion rates observed were higher than previously reported after RPV. Furthermore, it suggests that the booster dose might induce a T-cell response in previously unresponsive patients. The complete EPV and booster dose in patients under anti-CD20 and S1P modulators drugs resulted in a breakthrough infection rate similar to untreated patients.

## Supplementary Information

Below is the link to the electronic supplementary material.

Supplementary file1 (DOCX 26 KB)

## Data Availability

The data that support the findings of this study are available from the corresponding author, FL, upon reasonable request.
