# Correction: Evaluation of the efficacy of the SARS-CoV-2 vaccine additional and booster doses in immunocompromised patients with multiple sclerosis: the COVACiMS study

**DOI:** 10.1007/s00415-025-13494-2

**Published:** 2025-11-18

**Authors:** Filipa Ladeira, Claudia Nobrega, João Cerqueira

**Affiliations:** 1 Multiple Sclerosis Center of Integrated Responsibility, Hospital de Santo António Dos Capuchos, Unidade Local de Saúde São José, Lisbon, Portugal; 2 Centro Clínico Académico de Lisboa, Lisbon, Portugal; 3 ICVS/3B’s- PT Government Associate Laboratory, Life and Health Sciences Research Institute (ICVS), Braga, Portugal; 4 Neurology Department, Hospital de Braga and 2CA – Clinic Academic Centre Braga, School of Medicine, University of Minho, Braga, Portugal

**Correction: Journal of Neurology (2025) 272:288** 10.1007/s00415-025-12991-8

In the original version of this article, there was a misspelling in the transcription of an existing author’s name in the acknowledgement sections.

The author’s names currently displayed as

Carlos Capela, Cristina Araújo, João Sequeira, Marisa Brum, Stephanie Castro, Teresa Grine, João Canto-Gomes, Sara Silva-Ferreira, Catarina Ramos Fernandes, Irene Mendes, Dina Silva, Manuel Salavisa, Filipa Serrazinar, João de Sá, João Abreu, Maria José Sá, Rui Guerreiro, Ana Arraiolos.

but the complete and correct list of authors should be

Carlos Capela, Cristina Araújo, João Sequeira, Marisa Brum, Stephanie Castro, Teresa Grine, João Canto-Gomes, Sara Silva-Ferreira, Catarina Ramos Fernandes, Irene Mendes, Dina Silva, Manuel Salavisa, Filipa Serrazina, João de Sá, João Ferreira, Miguel Leal Rato, Miguel Schön, Joana Guimarães, Teresa Mendonça, Pedro Abreu, Maria José Sá, Rui Guerreiro, Ana Arraiolos.

In the Acknowledgements section, the following introductory sentence “COVACiMS Collaborators and Affiliations” was missing and should have been

COVACiMS Collaborators and Affiliations: Carlos Capela^1,3^, Cristina Araújo^8^, João Sequeira^1,3^, Marisa Brum^2,3^, Stephanie Castro^2,3^, Teresa Grine^1,3^, João Canto-Gomes^4^, Sara Silva Ferreira^4^, Catarina Ramos Fernandes^4^, Irene Mendes^6^, Dina Silva^6^, Manuel Salavisa^7,8^, Filipa Serrazina^7^, João de Sá^9,10^, João Ferreira^9^, Miguel Leal Rato^9,11^, Miguel Schön^9^, Joana Guimarães^12^, Teresa Mendonça^12^, Pedro Abreu^12,13^, Maria José Sá^14,15^, Rui Guerreiro^16^, Ana Arraiolos^17^

The original article has been corrected.

