# Supplementary material for: Evaluation of the efficacy of the SARS-CoV-2 vaccine additional and booster doses in immunocompromised patients with multiple sclerosis: the COVACiMS study
Source: J Neurol. 2025 Mar 25;272(4):288. doi: 10.1007/s00415-025-12991-8 (PMC11937187; doi:10.1007/s00415-025-12991-8)
Supplement: Supplementary file 1 — Supplementary file1 (DOCX 26 KB) [file 415_2025_12991_MOESM1_ESM.docx]

**Supplementary material**

**Data collection**

At enrollment, demographic and clinical baseline data were collected from patients’ records and through a structured questionnaire, including the following:

- date of birth;
- sex;
- COVID-19 infection occurrence;
- date and PCR confirmation prior to inclusion;
- first /second/third vaccine dose date and type (BNT162b2- BioNTech; mRNA-1273- Modern; Ad26.COV2.S- Janssen; ChAdOx1- AstraZeneca);
- MS type;
- date of MS first symptom;
- date of MS diagnosis;
- last EDSS evaluation;
- date of last EDSS evaluation;
- corticosteroid intake until 4 weeks prior to any dose of COVID-19 vaccine (yes/no);
- treatment characterization at the time of each COVID-19 vaccine dose:
  - DMT start date;
  - in patients under IRT/Natalizumab/anti-CD20 drugs date of last treatment before each COVID-19 vaccine dose and treatment interval considering the last 2 administrations (regular interval dosing vs extended interval dosing, and the number of weeks between treatments in the latter);
- ongoing treatment (yes/no and in the latter DMT end date);
- and blood sample date.

**Laboratory procedures**

1. Sample processing and isolation of peripheral blood mononuclear cells

Venous blood samples were collected into lithium heparin tubes and dry tubes. On the same day, samples were shipped from each participating hospital to the Life and Health Sciences Research Institute (ICVS) within a 22 ºC Temp Shell Element (DeltaT, Germany) to maintain samples between 18 to 22 ºC during transportation. Twenty-four to 36 h after collection, samples were processed. Blood in dry tubes was centrifuged, and serum was collected, aliquoted, and stored at -80 ºC until used for immunoglobulin quantification. A 100 μL blood aliquot was retrieved from the lithium heparin tubes for T and B cell enumeration (described below); T cell *Xtend* solution (Oxford Immunotec, UK) was added (25 μL per mL of blood) to the remaining blood on the heparin tubes and incubated for 15 min, at room temperature, before gradient centrifugation using Histopaque 1077 (Sigma-Aldrich, USA) to isolate peripheral blood mononuclear cells (PBMCs). PBMCs were recovered to AIM-V medium (Oxford Immunotec, UK) and, after being washed, 10 μL of the PBMCs suspension was used for leucocyte enumeration upon incubation with anti-CD45 Pe (clone HI30; Biolegend, USA) and acquisition on a MUSE cell analyzer (Millipore Corporation, USA). PBMCs were subsequently used for the ELISPOT assay.

2. T and B cell counts in whole blood

Blood B and T cell counts were determined using previously titrated anti-CD3 PercP-Cy5.5 (clone OKT3) and anti-CD20 Pe (clone 2H7) or anti-CD8 Pe (clone RPA-T8, all from Biolegend, USA) antibodies (2 antibody mixes were made). Upon a 15 min incubation, RBC Lysis/Fixation Solution (BD Biosciences, USA) was added, and cells were incubated for further 15 minutes before acquisition on a MUSE cell analyzer.

3. ELISPOT assay

For the enumeration of spike and nucleocapsid-reactive T cells producing IFNγ, the T-SPOT.COVID (Oxford Immunotec, UK) was used according to the manufacturer’s instructions. Briefly, 2.5 x 10^5^ PBMCs/well, per participant, were added to 4 wells of an anti-IFNγ pre-coated plate; 50 μL of ready-to-use stimuli was added to each well as follows: i) spike antigens; ii) nucleocapsid antigens, and; iii) phytohemagglutinin (PHA; positive control). Fifty microliters of AIM-V medium was added to the PBMCs for the negative control well. The plate was then placed on a humified incubator (37 ºC, 5% CO_2_) for 16 to 20 h . Afterward, the wells were washed with PBS to remove cells, and the detection antibody (alkaline phosphatase-conjugated) was added, followed by the substrate BCIP/NBT. For spot counting, each well was photographed using a stereomicroscope (Olympus SZX2-ILLT), and the spot forming units (SFU) were enumerated using the ImageJ software (National Institutes of Health, USA). According to the manufacturer’s instructions, an assay was considered valid whenever ≥20 SFU were counted on the positive control well and ≤10 SFU on the negative control well. A participant was considered to have reactive T cells whenever SFU on the spike and/or nucleocapsid wells (upon subtracting SFU of the negative control well) was >8. Data were represented as SFU/1 million PBMCs.

4. SARS-CoV-2 Serology

Serum aliquots from each participant were sent to a certified laboratory for quantification of SARS-CoV-2 spike-specific IgG (neutralizing antibodies; Liaison SARS-CoV-2 TrimericS IgG; DiaSorin Inc., USA) and SARS-CoV-2-nucleocapsid specific IgG (Anti-SARS-CoV-2 NCP ELISA IgG; Euroimmun, Germany). The result interpretation and detection limits of each test, according to the manufacturer’s instructions, can be found in Supplementary Table 2. Seroconversion was based a positive result on SARS-CoV-2 spike-specific IgG.

**Supplementary Table 1.** Vaccination scheme recommended in Portugal between January 2021 and September 2022.

|  | Primary vaccination course | | | | Booster dose |
| --- | --- | --- | --- | --- | --- |
|  | Baseline | Week 4 | Week 8 | ≥ Month 3 | ≥ Month 3 after completed primary vaccination/infection |
| General population^a)^ | mRNA | mRNA |  |  | mRNA |
|  | AZD1222 |  | AZD1222 |  |  |
|  | Ad26.CoV2 |  |  |  |  |
| Immunosuppressed subjects^b)^ | mRNA | mRNA |  | mRNA | mRNA |
|  | AZD1222 |  | AZD1222 | mRNA |  |
|  | Ad26.CoV2 |  |  | mRNA |  |
| If COVID-19 before primary vaccination |  | | | mRNA | mRNA |
|  |  |  |  | AZD1222 |  |
|  |  |  |  | Ad26.CoV2 |  |

1. Including non-treated multiple sclerosis patients and the ones treated with non- immunosuppressive disease-modifying therapies (interferon beta, and glatiramer acetate)
2. Including multiple sclerosis patients treated with other disease modifying therapies not specified in a).

**Supplementary Table 2.** Interpretation of result and detection range of the SARS-CoV-2 immunoglobulin quantification tests.

|  | **Nucleocapsid-specific IgG** | **Spike-specific IgG** |
| --- | --- | --- |
| **Cat no., Manufacturer** | EI 2606-9620-2 G, Euroimmun | 311510, DiaSorin |
| **Assay specifications** | ELISA; semiquantitative  Reported sensitivity: 94.6% (> 10 days after symptom onset our direct SARS-CoV-2 detection)  Reported specificity: 99.8% | Chemiluminescence; quantitative  Reported sensitivity: 98.7% (>14 days after COVID-19 diagnosis) or 99.0% (21 days after the first vaccine dose)  Reported specificity: 99.5% |
| **Result interpretation** | Negative: ratio <0.8  Borderline: ratio [0.8; 1.1[  Positive: ratio ³1.1 | Negative: <33.8 BAU/mL  Positive: ³33.8 BAU/mL |
| **Detection range** | n.a. | [4.81; 2080] BAU/mL |
| **Value attributed when outside detection range** | n.a. | Bellow: 4.60 BAU/mL  Above: 2100 BAU/mL |

AU, arbitrary units; BAU, binding antibody units; n.a., not applicable.
